# Analysis of Default Mode Network in Social Anxiety Disorder: EEG Resting-State Effective Connectivity Study

**DOI:** 10.3390/s21124098

**Published:** 2021-06-15

**Authors:** Abdulhakim Al-Ezzi, Nidal Kamel, Ibrahima Faye, Esther Gunaseli

**Affiliations:** 1 Centre for Intelligent Signal and Imaging Research (CISIR), Department of Electrical and Electronic Engineering, Universiti Teknologi PETRONAS, Seri Iskandar 32610, Malaysia; abduhalezzy@yahoo.com (A.A.-E.); nidalkamel2@hotmail.com (N.K.); 2 Psychiatry Discipline Sub Unit, Universiti Kuala Lumpur Royal College of Medicine Perak, Ipoh 30450, Malaysia; esther@unikl.edu.my

**Keywords:** effective connectivity network, partial directed coherence (PDC), social anxiety disorder (SAD), default mode network (DMN), electrophysiological biomarkers (EEG), resting state network (RSN), Granger causality (GC)

## Abstract

Recent brain imaging findings by using different methods (e.g., fMRI and PET) have suggested that social anxiety disorder (SAD) is correlated with alterations in regional or network-level brain function. However, due to many limitations associated with these methods, such as poor temporal resolution and limited number of samples per second, neuroscientists could not quantify the fast dynamic connectivity of causal information networks in SAD. In this study, SAD-related changes in brain connections within the default mode network (DMN) were investigated using eight electroencephalographic (EEG) regions of interest. Partial directed coherence (PDC) was used to assess the causal influences of DMN regions on each other and indicate the changes in the DMN effective network related to SAD severity. The DMN is a large-scale brain network basically composed of the mesial prefrontal cortex (mPFC), posterior cingulate cortex (PCC)/precuneus, and lateral parietal cortex (LPC). The EEG data were collected from 88 subjects (22 control, 22 mild, 22 moderate, 22 severe) and used to estimate the effective connectivity between DMN regions at different frequency bands: delta (1–3 Hz), theta (4–8 Hz), alpha (8–12 Hz), low beta (13–21 Hz), and high beta (22–30 Hz). Among the healthy control (HC) and the three considered levels of severity of SAD, the results indicated a higher level of causal interactions for the mild and moderate SAD groups than for the severe and HC groups. Between the control and the severe SAD groups, the results indicated a higher level of causal connections for the control throughout all the DMN regions. We found significant increases in the mean PDC in the delta (*p* = 0.009) and alpha (*p* = 0.001) bands between the SAD groups. Among the DMN regions, the precuneus exhibited a higher level of causal influence than other regions. Therefore, it was suggested to be a major source hub that contributes to the mental exploration and emotional content of SAD. In contrast to the severe group, HC exhibited higher resting-state connectivity at the mPFC, providing evidence for mPFC dysfunction in the severe SAD group. Furthermore, the total Social Interaction Anxiety Scale (SIAS) was positively correlated with the mean values of the PDC of the severe SAD group, r (22) = 0.576, *p* = 0.006 and negatively correlated with those of the HC group, r (22) = −0.689, *p* = 0.001. The reported results may facilitate greater comprehension of the underlying potential SAD neural biomarkers and can be used to characterize possible targets for further medication.

## 1. Introduction

Social anxiety disorder (SAD) is a prevalent psychiatric health condition identified by persistent panic and avoidance in a domain of social tasks that involve a possibility of scrutiny by others [1]. Cognitive models underline the discriminatory processing of interior and exterior threat signals that SAD individuals engage in when confronted with others (e.g., negative beliefs, attentional prejudice to disinclination faces [2] as an indicator of the expansion and preservation of the SAD [3,4]. Accumulating evidence from experiment-based functional magnetic resonance imaging (fMRI) studies revealed such biases in the findings of fronto-limbic disruptions. Generally, the electrocortical alterations were found in specific brain regions (e.g., anterior cingulate cortex (ACC), dorsolateral prefrontal cortex; dPFC) [5,6]. A previous systematic review of SAD indicated that adolescents and children with SAD exhibit emotional dysregulation such as social avoidance, more safety behaviors, recurrent negative thoughts, biased perception and awareness of social information, and deficiencies in emotional expression.

The SAD’s neurobiological effects have been confirmed by baseline resting-state fMRI studies, which allow substantial (instantaneous, task-independent) neural networks to be examined. Studies have revealed aberrant interactions in the SAD within spatially distant regions, indicating that the dysfunction includes a disturbance of the baseline in decentralized neural systems. For example, a reduced frontal limbic functional connectivity (FC) and aberrant effective connectivity (EC) were observed in SAD groups compared with HC groups [7,8]. Additionally, in SAD patients, significant variations in task-positive and task-negative networks (e.g., attention network, DMN, visual networking system) were observed [9]. Overall, the fMRI results suggest abnormal resting-state behaviors, and these behaviors can reflect a threat-sensitive network in SAD. However, the current findings of neural networks in the brain are restricted in fMRI owing to its low temporal resolution. Thus, fMRI does not detect the millisecond to second timescales due to its slow variance in the hemodynamic response.

Another more effective neuroimaging approach is EEG, which assists in evaluating the rapid and lagged instantaneous brain activation in both time and frequency scales, so that actual neurobehavioral networks can be identified [10]. While various concepts of positive and negative impacts in SAD have been posited, less is understood about how SAD is being interpreted at the neural level. One approach for addressing this problem is to investigate how neural brain activity in SAD communicates using EEG during resting-state in DMN regions. To date, EEG has hardly been applied in research on the EC of SAD at rest. In several studies, the EEG method was employed to investigate SAD’s cognitive models and behavioral mechanisms in social engagement tasks, visual tasks, and DMN, for review [11]. 

Among the resting-state elements, the DMN received great attention nowadays [12,13]. DMN has latterly protruded as an important scientific subject for the researchers as well as for clinical application because it shows distinctive features in the resting-state [14]. Science suggests that the DMN is capable to contribute to the psychological exploration of emotional and social functionality, which can help to study the neural symptoms of SAD. The DMN is perceived to be partially responsible for the intestinal consciousness. DMN is localized in three regions; precuneus/PCC, LPC, and mPFC [12]. The DMN has exhibited higher activation in the resting-state when a person becomes more concentrated internally rather than externally or on their internal mental-state processes, such as self-referential processing, theory of minds, autobiographical memory retrieval [15]. Neural changes within DMN have been found in different mental brain disorders such as Alzheimer’s disease [16], consciousness disorder [17], and major depressive disorder [18]. SAD individuals have exhibited significant enhancements in the interconnections in the cortical brain, mainly in the precuneus, which is an essential component of the DMN [19]. Additionally, SAD patients exhibited significant correlations with the substantial regions involved in the DMN during the expectation, emotional excitement, awareness regulation, and reception of monetary gain [20,21]. The left precuneus and left supramarginal gyrus in the DMN have exhibited an enhanced FC in SAD individuals, compared with the HC group [22,23]. 

The main objective of our study is to estimate the severity of SAD by categorizing its grades (severe, moderate, mild, and HC) using effective connectivity (PDC algorithm). Though FC has the ability to determine the brain connectivity between brain regions (e.g. coherence), it fails to present the directions and amount of information flow between brain regions. Therefore, PDC overcome that limitations and by revealing the direct information flows between brain areas independent of the influence of other areas. PDC is a frequency-domain metric based on the Granger causality approach [24], a method that estimates the directed information flow between two signals while neglecting the volume conduction (insensitive to noise). Due to the high temporal resolution, EEG provides us with information about the electrocortical process in the brain during the DMN-resting-state which assists in quantifying the EC among DMN regions.

To the best of our knowledge, this was the first DMN analysis in which the severity of SAD (severe, average, mild, and control) was evaluated by using the effective connectivity measures (PDC) to assess effective network characteristics at different frequencies (delta, theta, alpha, low beta, and high beta). Our initial research hypothesis was whether SAD is correlated with disrupted neural activation in DMN processing. It was hypothesized that the four SAD groups would respond differently in the DMN resting state, stimulating brain interactions between various brain regions and enhancing the EC of the brain cognitive network. The spectral EC was used to characterize the directions, power intensity, and influences. 

The following are presented in the remainder of this paper: materials and methods (for data acquisition), statistical dimensions, results (scientific findings), discussions, limitations, and potential directions with conclusions.

## 2. Materials and Methods

### 2.1. Participants

Eighty-nine (89) participants were chosen from 417 respondents (34 females and 54 males; 17–25 years old (mean (M) = 22.35, standard deviation (SD) = 0.98) (M = 23.16, SD = 0.85)) who submitted SIAS self-assessment reports. The adequate sample size was defined by performing a power analysis reported in [25]. Respondents of both genders were enrolled in the experiment to generalize the study’s findings and validate the findings of our study. Table 1 shows the demographics data and group characteristics. Depending on the SIAS scores, the participants were divided into four categories: the control group (SIAS score < 20), mild group (SIAS score < 35), average group (SIAS score < 50), and severe group (SIAS score ≥ 50). The selected participants have been diagnosed with SAD using the DSM-IV-based Composite International Diagnostic Interview [26]. One (1) participant was excluded because of data-collection issues. None of the subjects had a history of psychological, neurological, or surgical deficiency that may have affected their brain activity or metabolic processes. At the time of recruitment and during the EEG session, no subjects had received any pharmacological or psycho-therapeutic treatment. All the recruited subjects were given a single sheet containing both information about the research and a waiver of written informed consent, and they were compensated for their time and cooperation. The experiment complied with the Helsinki Declaration [27]. The protocol for the study was carefully reviewed, accepted, and approved by the Medical Research Ethics Committee of the Royal College of Medicine Perak, Kuala Lumpur University.

### 2.2. Experimental Design

The rest-state recordings were conducted in an EEG laboratory. The participants were asked to sit comfortably, keeping their eyes closed, in a comfortable, semi-darkened dim room. The subjects were requested to abstain from consuming alcohol and caffeine prior to their EEG recording (for at least 5 h). The subjects were instructed to habituate themselves to the procedure of the EEG recording task prior to the experiment. The participants were then provided with a description of the EEG protocol and signed an informed consent form. After the electrodes were mounted on the scalp, the EEG protocol was started to acquire the resting state of the EEG for 5 min (eyes closed). The participants were informed to close their eyes, remain calm, and let their minds wander freely. Eventually, all the participants answered the self-report questionnaires and were debriefed.

### 2.3. EEG Data Acquisition, Preprocessing and PDC Implementation

**Figure 1:**
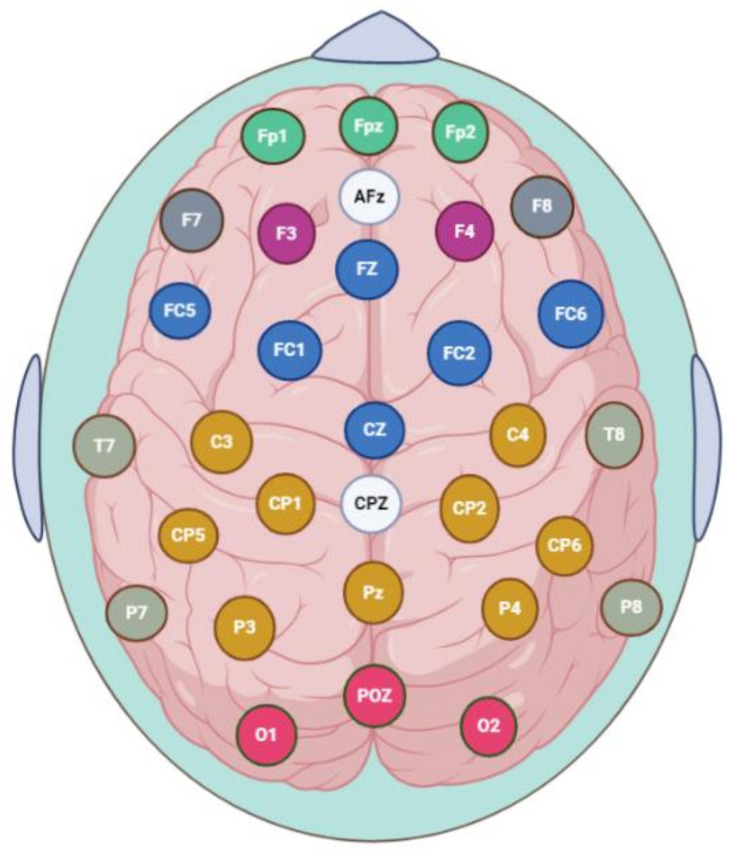
Topographical placement of 32 electrodes using the extended international system 10–20, indicating the distribution of the electrodes on the cortical scalp, categorized as follows: prefrontal (Fp1, Fp2), medial prefrontal—mPFC (Fpz), ventrolateral prefrontal (F7, F8), dorsolateral prefrontal (F3, F4), frontal (FC5, FC1, FC2, FC6), midfrontal (Fz, Cz), temporal (T7, T8, P7, P8), parietal (C3, C4, CP5, CP1, CP2, CP6, P3, P4), midparietal (Pz), occipital (O1, O2), and midoccipital (POz). The system is referenced to CPz, and grounded at AFz.

Scalp EEG signals were continuously acquired during a 5-min baseline (3 s eyes closed) duration using a referential 32-channel shielded cap while the participants were seated (ANT Neuro, Enschede, The Netherlands), as shown in Figure 1. All 32 gel-based sensors were attached to an EEG head eegosports cap (ANT Neuro, Enschede, The Netherlands), referenced to CPz, and grounded at AFz, and all signals were digitally offline re-referenced to the common average reference. The impedances were kept below 10 kΩ. 

Recorded electrophysiological data from every channel were digitized at a sampling rate of 2048 Hz. Raw EEG signals were preprocessed offline to eliminate unnecessary data (noised segments) using BESA Research Toolbox 6.0. To eradicate the high-frequency electrocortical artifacts, signal contamination, and low-frequency deflections, we applied a FIR bandpass filter to obtain the optimal segments between the low and high frequencies (0.4–50 Hz) [28,29]. Artifacts such as blinking, horizontal and vertical eye motions (HEOG and VEOG, respectively), breathing, power interference, and cardiac movements were visually inspected and automatically discarded using BESA-based artifact-based spatial detection and brain signal topography [30]. The data then were down-sampled to 256 Hz. The open-source toolboxes also include EEGLAB for the visualization of topographic maps [31]. According to the standardized frequency bands, the data observations and segments were divided into the following ranges: delta (1–3 Hz), theta (4–8 Hz), alpha (8–12 Hz), low beta (13–21 Hz), and high beta (22–30 Hz). To prevent non-stationary confusion, we segmented the data into approximately stationary nonoverlapping short time-series data (4 s). The total quantity was 29 epochs (116 s), which was within the domain of a previous resting-state analysis [32], to achieve a balance between the stationarity and the model order fit, as longer time series support more accurate parameter estimation for locally appropriate linear autoregressive models. For our data, we found that shorter segments (1–2 s) negatively affected the model fit for many segments [33]. The EC was calculated based on the exact low-resolution brain electromagnetic tomography (eLORETA) localized EEG data (Section 2.4) for each segment (4 s epoch) and then averaged to have one PDC matrix for every subject with 8 (channels) × 8 (channels) × 5 (bands). The implementation of PDC algorithm is reported in Appendix A.

### 2.4. EEG Source Localization-Based Effective Connectivity

**Figure 2:**
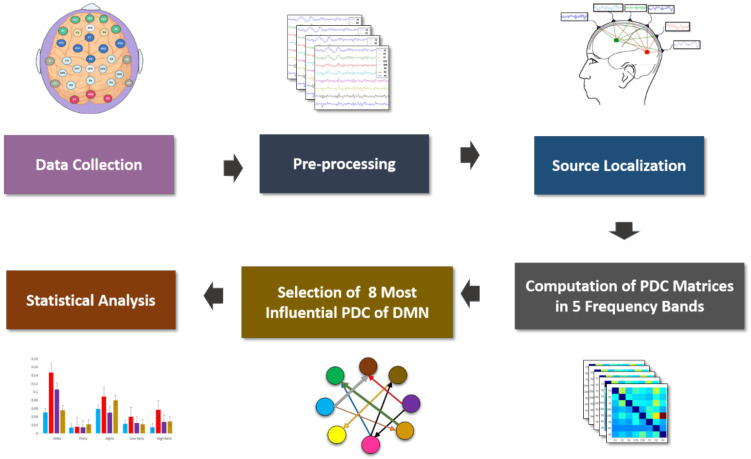
Block diagram for the EEG data analysis module to identify the parameters of the effective connectivity network between default mode network (DMN) regions.

The acquired EEG signals can be used to perform the inverse problem to define the locations of the predominant sources of the brain activity. To provide more validity to this approach, an additional source localization analysis of all frequency oscillations (0.4–50 Hz) in resting-state was performed using eLORETA in search of the active sources generating the scalp potentials [34]. The source localization analysis was performed using nonparametric statistical mapping methodology supplied by the eLORETA software. The eLORETA mechanism is a discrete, three-dimensional distributed, linear, weighted minimum norm inverse solution and has the ability to reconstruct intercortical activity with correct localization from scalp EEG data [35,36]. Additional merit of eLORETA is that it has no localization bias even in the existence of noise [37]. Generally, the source model of eLORETA estimates the information about the orientation of dipole sources. In this work, we applied a moving window with 1 s (time scale) to strengthen the revelations of modulations in different cortical regions, as reported in earlier MEG and EEG works [38,39]. The comparison between SAD groups and HCs were performed on log-transformed data using the non-parametric statistical mapping methodology supplied by the eLORETA software package. Latter, the time source electrical potentials acquired from the localized EEG signal waveforms were then exported as mean activity in the EC analysis. Through our analysis, it has been found that a set of eight operationally active cortical areas indexed by four distinct: PCC/Precuneus (PZ, P3 and P4), mPFC/vmPFC (FZ, F3, and F4), and LPC (Angular Gyrus and Supramarginal Gyrus (CP5 and CP6)) [40,41,42,43,44], is dominant and active in all subjects more than the other cortical areas of the brain. Then, the intercortical surfaces were parcellated into 15,000 anatomical vertices on the basis of Montreal Neurological Institute (MNI) templates and Talairach coordinates [28,45]. We have excluded the non-active cortical areas from any further EC analysis. Table 2 shows the most active EEG regions which could, in large, account for DMN network. EC weights between these active regions were calculated for each artifact-free EEG segment in the mentioned frequency bands. For each subject included in our analysis, we repeated the foregoing procedure to produce the final average PDC for each group from the localized data. Throughout the analysis, the optimum model order for each subject from Akaike information criterion (AIC) varied from 4 to 10 [46]. The averaged EC (PDC values) for all pairwise region of interests (ROIs) of all the frequency wave connections in the SAD groups exhibited the pairwise relationship between any two ROIs within the network. The entire process is shown in Figure 2.

### 2.5. Clinical Assessment

SIAS reveals and quantifies the approximate fears of more general social interaction and is in accordance with the Social Phobia-Circumscribed DSM-III-R definitions. The SIAS scale has been shown to have high internal consistency levels and reliability for testing-retests. SIAS may distinguish between social phobia, agoraphobia, and simple samples of phobia, as well as between social phobia and common samples [55].

The scale is found to be changed with medication and stabilize over no-treatment. Furthermore, the SIAS scale tends to be accurate, useful, valid, and easily scored for clinical and research applications, because it exhibits an improvement over existing SAD measures [56]. The SIAS measure is intended to recognize two distinctly different aspects of SAD, including apprehension, escaping daily social interactions, internal fear, and avoidance of performing experiences linked with the perception of sociscrutiny (e.g., dining, consuming food and drinks in public, and writing in the company of others) and more general engagements (e.g., attending or organizing events or making new friends). Indeed, a recent systematic review [57] revealed that the SIAS scale has obtained higher positive scores for psychometric accuracy than other social evaluation measures. Four subdomains (control, mild, average, and severe) were measured with 20 questions. The total scores in each subdomain ranged from 1 to 4, with higher scores exhibiting a higher severity of SAD [58].

### 2.6. Statistical Analysis

The distribution of the information flow measurements was assessed with a univariate ANOVA test, and the statistical findings are reported by F values and significant values (*p*). All the statistical findings were presented as the mean ± standard deviation. To examine the group differences before conducting the ANOVA test, we used the multi-sample Kolmogorov–Smirnov test to check if the data were normally distributed [59]. We applied the expansion of the Bonferroni test (α = 0.05) to correct for multiple comparisons. The analysis of mean variances in our study included two independent variables (Group: severe, moderate, mild, and control) (Regions; F3, F4, FZ, CP5, CP6, P3, P4, and PZ) and one dependent variable (PDC values)); therefore, a one-way univariate ANOVA and Tukey’s HSD post hoc test for different comparisons (*p* < 0.05) were performed to evaluate the main differences between the causal information flow of frequency EEG data of SAD groups. The ANOVA test was applied within subject factor, i.e., we made a comparison between the same frequency band (e.g., delta) from all SAD conditions. The relationship between the SIAS scores and PDC values (PDC values and SIAS scores) in all frequency bands was investigated by Pearson’s correlation test [60]. SPSS software (version 25.0.0.0, IBM Corp., Armonk, NY, USA) was used for all statistical analyses.

## 3. Results

The experimental findings are presented in the following three subsections: the subjective analytics, the EEG-based EC metrics, and the correlation analysis between the DMN values and SIAS scores.

### 3.1. Subjective Data Analysis

Initially, we examined the self-report questionnaire data to determine the group differences between the 4 groups. The total calculated percentages of the SAD groups—control, mild, average, and severe—were 18.38%, 27.09%, 26.12%, and 28.38%, respectively. The participants’ responses in the questionnaires were subjected to analysis of variance (ANOVAs) and a nonparametric test (Mann–Whitney) for parameters that were not normally distributed. However, the groups did not differ significantly with regard to age, F (1, 87) = 2.664, *p* = 054, η2 = 0.093. Compared to those of all other groups, severe participants exhibited significantly higher SAD scores during the experiment: F (1, 87) = 21.06, *p* = 0.001, η2 = 0.53.

### 3.2. Power Analyses 

The averaged resting-state EEG power across all active sources (ROIs) was examined with 4 SAD groups (severe, moderate, mild, and HC) × 4 frequency bands (delta, theta, alpha, and beta) using one-way ANOVA with repeated measures on the last factor. There was no significant effect for group or frequency band (all *p* > 0.05) and no group frequency relationship was found (*p* = 0.134). The ANOVA test evidenced significant emotional state effects with absolute alpha power F (1, 119) = 4.23, *p* = 0.023, η2 = 0.21, and delta power F (1, 119) = 2.533, *p* = 0.012, η2 = 0.22. Figures S1 and S2 (Supplementary Materials) shows the averaged absolute power of delta, theta, alpha, and beta, along with topographies for different SAD severities and HC groups.

### 3.3. Effective Connectivity in Different Frequency Bands

**Figure 3:**
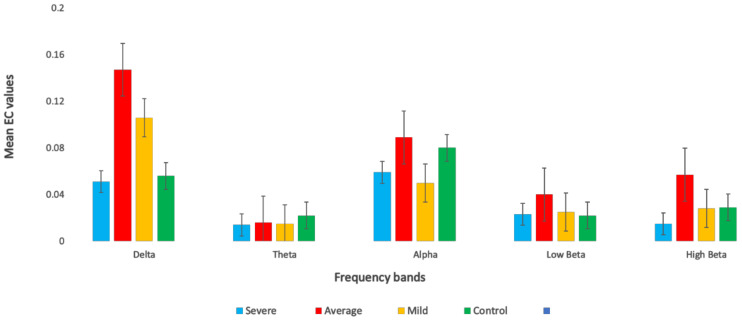
Average values of effective connectivity (EC) across different frequency bands in all SAD patients and healthy controls (HCs).

In the first experiment, EC was calculated for the control, mild, average, and severe SAD groups and averaged within the subjects of each group. Then, EC values were decomposed into different frequency rhythms (delta, theta, alpha, low beta, and high beta). The results in Figure 3 indicate significant differences between the four SAD groups in the delta and alpha bands: F (3, 252) = 3.937, *p* < 0.009, η2 = 0.1 and F (3, 252) = 3.766, *p* < 0.01, η2 = 0.1, respectively. In contrast to the alpha and delta bands, no significant differences were observed in the high beta (F (3, 252) = 1.571, *p* < 0.196, η2 = 0.04) and low beta (F (3, 252) = 0.410, *p* < 0.746, η2 = 0.04) bands. In the delta band, post hoc testing revealed significant 0.1466, SD = 0.3057) and mild (M = 0.1064, SD = 0.1586). The control and severe groups exhibited less effective connectivity of (M = 0.0563, SD = 0.0512) and (M = 0.0514, SD = 0.1052), respectively in the high beta (F (3, 252) = 1.571, *p* < 0.196, η2 = 0.1) and low beta (F (3, 252) = 0.410, *p* < 0.746, η2 = 0.1). This suggests that there were stronger connections among the DMN regions in the average and mild groups compared with those in the severe and control groups. Table 3 shows the ANOVA test between the SAD groups and HCs.

Moreover, in the alpha band, post hoc testing revealed significant differences in EC strengths between the groups; severe (M = 0.0587, SD = 0.0466), average (M = 0.0776, SD = 0.0707), control (M = 0.0797, SD = 0.0641), mild (M = 0.0501, SD = 0.0529). This indicated a stronger alpha connection in severe, average, and control groups more than in the mild group. Furthermore, the results indicated significant differences in the theta band between the severe and HC groups for F (3252) = 2.389, *p* < 0.05, η2 = 0.1, (M = 0.0141, SD = 0.0098), (M = 0.0216, SD = 0.0209), respectively. However, the results revealed no significant difference between the rest of the groups, F (3, 252) = 2.389, *p* < 0.069, η2 = 0.1, average (M = 0.0163, SD = 0.0024), and mild (M = 0.0147, SD = 0.0024). As mentioned previously, the high beta and low beta did not exhibit any significant differences between any groups, F (3, 252) = 1.571, *p* < 0.196, η2 = 0.1), F (3, 252) = 0.410, *p* < 0.746, η2 = 0.1). In the high beta band, the mean EC values were as follows: severe (M = 0.0242, SD = 0.0200), average (M = 0.0371, SD = 0.0515), mild (M = 0.278, SD = 0.034), and differences between the two different groups of average (M = control (M = 0.291, SD = 0.0356). In the low beta band, the mean EC values were as follows: severe (M = 0.23, SD = 0.0172), average (M = 0.251, SD = 0.0286), mild (M = 0.254, SD = 0.0244), and control (M = 0.022, SD = 0.0178). Clearly, the precuneus region exhibited the strongest EC among the DMN regions.

**Figure 4:**
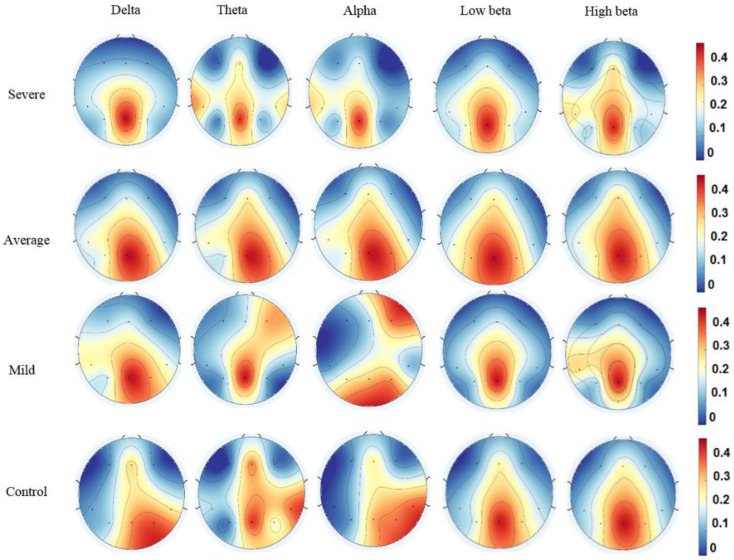
Topological maps of the mean total information intensity for all four SAD groups in the frequency bands. Red indicates greater PDC values; blue indicates a smaller PDC.

Topographic mapping of averaged mean EC values at various DMN regions during the resting-state is shown at different EEG bands in Figure 4. The results indicated greater EC values in the alpha and delta frequency bands in DMN regions compared with those of the other frequency bands for all four groups. Among the DMN regions, the delta and alpha bands were higher in the LPC and precuneus than in the other DMN regions, in agreement with previous findings. Therefore, the PCC/precuneus is suggested to be a major source hub that contributes to the function and mechanism of cognitive exploration and emotional states of SAD. Apart from that, whole brain connectivity was calculated for more validity, the DMN regions were more active than the others in all frequency bands (Figure S3, Supplementary Materials).

### 3.4. Pairwise EC of DMN Components in SAD

**Figure 5:**
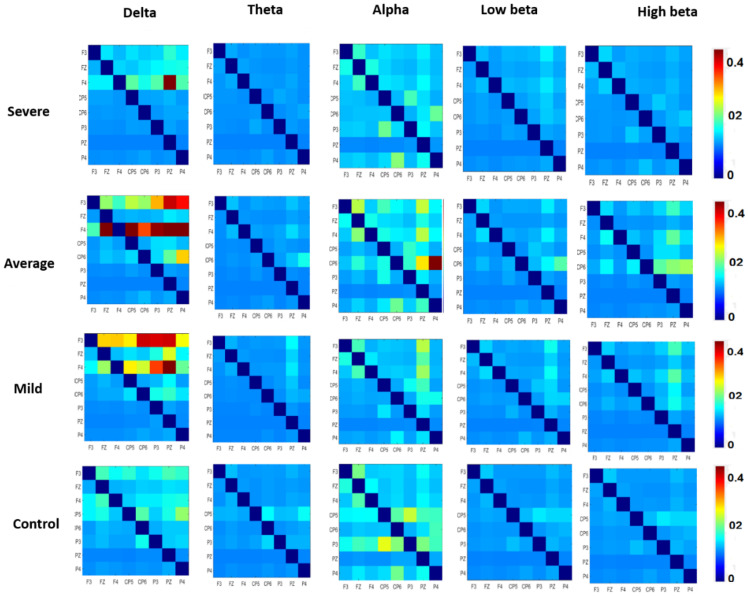
Mean EC intensity matrices of eight DMN neuronal clusters in the resting state. Every component in the matrix represents the mean EC magnitudes for all participants. The information flow can be represented by the flow from the lower rows to the left columns. The red color of the components reflects greater EC significance of the connectivity. Diagonal values are set as zero.

In this section, the heat-map of the average EC over each group is constructed. This map is represented in matrix form, where the entry (i, j) indicates the causal influence of the j-region on i-region at a certain band. The values of the heat map are displayed in a color scale, where blue represents the minimum range of the EC values, and red represents the maximum range. In the delta band, the severe group exhibited more information flows in the right hemisphere (LH) than in the left hemisphere (RH) between the precuneus sides and mPFC; the LPC and mPFC (PZ→F4); (CP5→F4), from precuneus where the mPFC only receives the information, as shown in Figure 5. Greater information flows in the RH compared with the LH were detected in the LPC and right mPFC regions (P4→F4 and PZ→F4), right LPC (P4→CP6), and mPFC (FZ→F4). In the case of the mild group, enhanced information flow in RH was higher than that in LH between the precuneus sides (F3, PZ and P4), and (PZ→F3 and P3→F3); (F4→F3 and (P4→F4). HC exhibited greater information flow from RH to LH (P4→CP5). Additionally, in the alpha, there were more significant differences in EC in the RH than in the LH with long-domain correlation in the precuneus and right LPC regions for the average and mild groups (PZ→F4, FZ→F4 and P3→CP6): (PZ→F3, PZ→F4 and PZ→CP6), whereas the HC group exhibited a larger quantity of causal information flow in the LH than in the RH in the left LPC and mPFC sites (CP5→P3, FZ→F3 and P3→CP5).

In comparison, smaller amounts of information flow within the left LPC region (CP5→P3 and P4→CP6) were observed for the severe group. However, the theta band exhibited a smaller flow of information between the precuneus and the mPFC (PZ→F4) and LPC (CP6→P3) for the HC group than for the severe group. Compared with the average group, mild participants exhibited a greater flow of information between the precuneus, LPC, and mPFC (P→F4, PZ→F3, and PZ→CP5). Within the high beta band, the HC group exhibited greater information transmission between the LPC and the precuneus regions (PZ→CP5) in the LH. Thus, HC subjects experienced more robust EC than the severe group. In addition, for the average group, there was enhanced information flow in the RH relative to the LH at the precuneus and rLPC (PZ→CP6, P4→CP6). 

**Figure 6:**
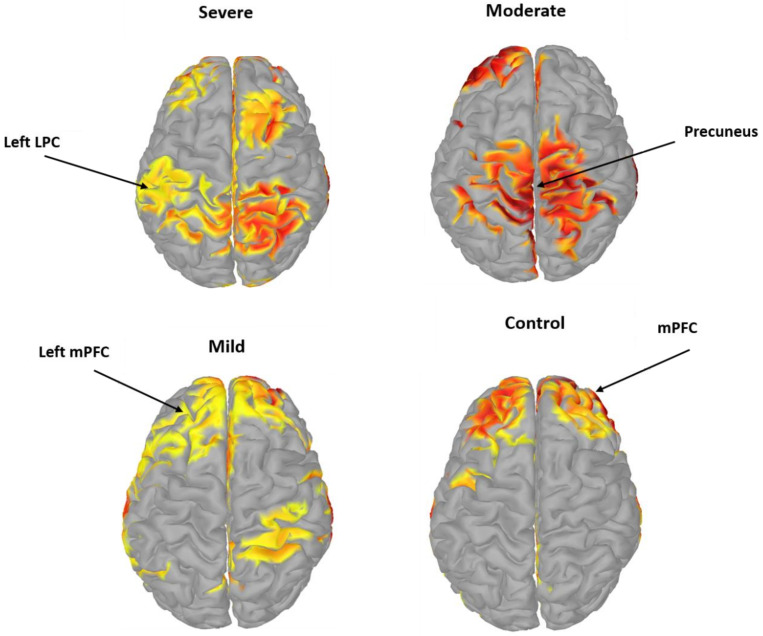
A 3D visualization of the computed EC source from EEG activity at the alpha band. It represents the highest 20% values at a threshold of significant level *p* < 0.05. It represents EC for severe group (top left), moderate (top right), mild (bottom left), and control (bottom right).

Similarly, for the mild class, there was a significant amount of information flow in the RH (PZ→F4). Lastly, the high beta band exhibited greater information flow than the low beta band. Firmly, the changes in EC between the DMN regions in SAD patients suggested enhanced regulation by the neural system of SAD, compared with the findings for the HC group. These results indicate that the abnormal EC between the DMN regions was related to a brain-function change in SAD. Figure 5 illustrates the significant differences in the average absolute EC in both brain hemispheres for ROIs paired connections. Figure 6 has proven that the precuneus is the pivotal hub of the DMN in severe and moderate SAD groups compared to HC and mild groups. HC individuals have shown higher EC in the mPFC region compared to that of the other groups, which indicates higher cognitive functions. Mild and moderate groups have shown greater information flow in the left mPFC than the other groups. The data shown in Figure 6 is obtained from the alpha band due to its ability to explore mental illness and emotional states.

### 3.5. Correlation Analysis between EC Values and Self-Report Measures

**Figure 7:**
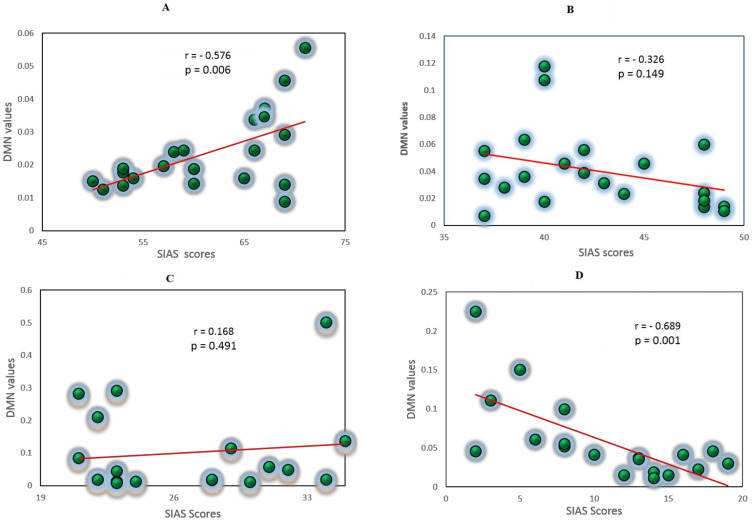
Correlation between the DMN-EC values and Social Interaction Anxiety Scale (SIAS) scores for the SAD groups, severe (**A**), moderate (**B**), mild (**C**), and control (**D**). Each circle represents a subject (participants).

The Pearson correlation coefficient was used to evaluate the linear correlation between the total averaged EC connectivity values (PDC values) in the DMN and the self-report questionnaire SIAS. Behaviorally, the severe group exhibited a positive significance correlation between the DMN and SIAS scores, i.e., r (22) = 0.576, *p* = 0.006, as shown in Figure 7. The correlation between the DMN and SIAS scores was negative for the control group, i.e., r (22) = −0.689, *p* = 0.001. The mild and average groups did not exhibit any significance in the correlation, r (22) = 0.168, *p* = 0.491, r (22) = −0.326, *p* = 0.149, respectively. Figure 7 show all the correlations between the SAD groups in the alpha band, due to its influence in brain disorders. Additionally, we have computed a correlation between DMN values and SIAS scores without dividing patients into different SAD conditions, as shown in Figure S4 (Supplementary Materials). The analysis showed significant negative correlation r (88) = −0.261, *p* = 0.006.

## 4. Discussion

To the best of our knowledge, this was the first study in which EEG-based EC was used to segregate the severity of SAD by categorizing its grading (control mild, average, and severe) and investigate the neurocorrelates characteristics of the DMN resting-state and the topological organization of the effective brain connectivity network in SAD. The results in Section 3 indicate significant values for EC in all the SAD groups in the dominant frequencies at rest, i.e., the delta and alpha bands. This finding agrees with previous studies on the DMN [61]. In the theta band, the results exhibited enhanced frontal midline interconnectivity and greater causal effects for the control group than for the severe group at the precuneus and LPC. However, in the theta band, we observed a tendency of enhanced frontal midline neuroconnectivity and greater causal connections in severe SAD and HC relative to those of the average and mild groups. Additionally, DMN connectivity amplitudes were negatively correlated to the SIAS state anxiety within the control group. SIAS scores of the individuals with SAD exhibited a positive significant correlation with the mean DMN values. We observed a significant difference in the effective connectivity intensities for all the SAD categories at the low frequency band (delta).

The ANOVA test in the ROI analysis revealed significantly greater EC values in the precuneus than in all other ROIs [62]. Moreover, the findings revealed that the precuneus was more active when individuals with SAD were in the resting-state (absent of any external stimulus), and it is implicated in a neural network of self-awareness, autobiographical tasks [63], cognitive interpretation, and future planning [64]. Thus, our study provided empirical evidence that precuneus dysfunction in SAD yields an imprecise interpretation of the inner emotional status as well as deficient processing of inner self-representation [65]. We believe that dysfunction of precuneus in the DMN of SAD individuals may be related to the development of SAD symptoms as well as to the high-level maintenance of self-focused attention [66]. Precuneus activity is enhanced during self-processing and perception (resting state) and reduced during goal-directed, non-self-referential tasks (performing task) [67]. Reduced activity in DMN brain areas (which was observed more frequently in our SAD subjects compared with HC individuals) may indicate a continued need to maintain self-focused awareness during a resting-state, presumably owing to the utilization of higher motivational pertinence to the anticipation of the next tasks in our experiment. 

Interestingly, statistical findings revealed that relative to the HC group, the SAD patients exhibited differences in EC neurocorrelates in the mPFC. The right mPFC activation was higher for the severe group. In comparison to severe group, the HC group exhibited a stronger correlation between the precuneus and the mPFC. This suggests that the changes in EC between the precuneus and the mPFC may have had a structural basis. Additionally, the severe group exhibited stronger causal activation in the mPFC and greater inhibition in the LPC in the DMN compared with the HC group. Previously, SAD patients exhibited increased activity within the left mPFC when performing a mental arithmetic task [68]. Our findings provide evidence of mPFC dysfunction in SAD patients and suggest that mPFC dysfunction may contribute to disparities within SAD categories. Neuroscientists believe that the increased inhibition of mPFC may reflect aberrant regulation of the neural mechanism in patients with SAD [69]. In the antecedent studies, the activity in the mPFC of SAD subjects had inconsistent findings about the neural activation (increased or decreased) in the resting-state but exhibited a significant difference in the performance of the cognitive tasks in the DMN areas [13,65]. The activity of the mPFC possibly reflects the neurocorrelates between cognitive behavioral processing and emotional states in positron-emission tomography [12,70]. Our results, together with the hypotheses on mPFC, indicate that mPFC plays a significant role in the influential psychopathology mechanism of SAD. Therefore, the mPFC may be engaged in the internal reflection of the social world [71] and plays a major role in the capacity to interpret certain mental states (e.g., cognitive function) [72]. The mPFC-precuneus dysfunction in this finding may represent the dysfunction of cortical regions that directly contribute to the pathophysiology of SAD. Our findings on the mPFC have not been reported previously. 

While many studies have revealed an aberrant resting state EC in different distant brain networks in SAD, to the best of our knowledge, no resting-state EEG study has been performed on the severity of SAD in DMN using EC. Our results also indicated that the EC of the mPFC regions in the DMN involved in self-referential and emotional processes exhibited stronger connectivity in the severe group, whereas the LPC sites of the DMN which were implicated in episodic memory and visual processing exhibited stronger connectivity in the HC group compared with the severe group. These findings are consistent with previously reported results of LPC and DMN research [73]. Dissociation between the mPFC and cortical midline LPC regions leads to the possibility of a behavioral differentiation within the DMN in terms of self-referential functions and contributes to the comprehension of perceptual and affective changes in SAD. The LPC exhibited a reduced and anomalous interaction in SAD patients compared with that of the HC group, suggesting greater behavioral avoidance for SAD participants, as reported in [73]. These behaviors have been proven to intermediate introspection and reflection upon one’s own mental state in HC [74]. Thus, we consider that increased inhibition of the precuneus-mPFC for the severe group can be linked to the deviant regulation of fear, whereas the reduced inhibition of the precuneus-LPC for the HC is linked to the impairment of autobiographical memory, as reported in previous work [69]. Table 4 presents the most relevant literature to our study. Compared to the existing literature about DMN activities in SAD individuals, our study is the first to investigate the severity of SAD (severe, moderate, mild, and HC) with a sufficient dataset (88 subjects) and exact localization of EEG sources. Our findings are in line with many fMRI studies that showed aberrant connectivity between DMN regions in resting-state.

This study had limitations. First, SAD participants were adults, and the comorbidity of other possible psychiatric disorders was not identified. The existence of comorbidities may have affected the findings, which requires further investigation. Second, the findings are focused on the SAD population and should not be extended to other psychological disorders or internalization issues. Third, it has also been reported that EC [78] cannot capture inhibitory connections with the same accuracy as excitatory connections, and was not accurate for discriminating between mono- and polysynaptic interconnections; thus, the findings reported herein may be validated with other connectivity algorithms. Fourth, we only concentrated on EEG signals in DMN areas; future EC research should focus on different brain modalities and regions, such as the fMRI subcortical regions. Fifth, we have used the 32-electrode EEG system to reconstruct brain activity (scalp potentials) in the brain cortex. Future research should focus on a high-density EEG system (i.e., 64, 128, or 256 electrodes) and compare the results with our findings. We also recommend researchers using effective connectivity features along with deep learning or machine learning models to detect the severity of SAD for clinical applications. Future research is recommended to employ fused brain imaging techniques to segregate the severity level of SAD (e.g., combing EEG model with fNIRS). The fusion between two different neuroimaging models is found to reflect better temporal and spatial characteristics of brain connectivity networks [79]. Finally, our research was a cross-sectional analysis; thus, only the resting state was investigated.

## 5. Conclusions

This study was the first investigation of the DMN EC network for SAD in different frequency bands. The use of directed EC estimates was suggested, and networks were identified via PDC analysis to evaluate the effect of SAD in DMN brain regions. Therefore, our findings indicated that individuals with SAD not only had abnormal alteration processing of certain socially anxious triggers, but also exhibited strong disturbance emotion processing in the basic nervous system pathway. The results reported herein are useful for the development of cognitive therapy models and the treatment of SAD. We reported a discriminatory deterioration or abnormality in the neural activity of the precuneus, mPFC, and LPC in severe SAD patients. It is believed that these regions with abnormal activations are biomarkers of SAD, representing the rudimentary pathophysiology and deficiency in the SAD groups. Overall, the subjective and electrophysiological findings indicate that EEG observations of the effects of SAD on emotional and cognitive processes in the DMN can serve as early biomarkers for SAD diagnosis and treatment.

## Figures and Tables

**Table 1:** Demographic data and group characteristics.

Group	Number of Participants		Total	Age		SIAS Score	
	Female	Male		Female	Male	Female	Male
Severe	12	10	22	22.13 ± 2.78	23.11 ± 1.02	67.53 ± 6.21	66.81 ± 5.32
Moderate	7	15	22	21.98 ± 3.11	22.21 ± 1.25	55.7 3± 7.81	54.41 ± 6.61
Mild	12	10	22	22.61 ± 2.32	21.71 ± 2.31	38.32 ± 512	37.71 ± 5.81
Control	8	14	22	21.76 ± 1.73	23.62 ± 1.65	14.71 ± 6.74	16.61 ± 7.34

**Table 2:** EEG electrodes, ROIs, default mode network region underneath, and MNI coordinates with Brodmann areas (BA) and functions.

ROI	MNI Coordinates			Anatomical Regions	BA	Function
	*x*	*y*	*z*			
FZ	0.6	40.9	53.9	Central mPFC	8,9,10	Attention [47]
F3	−35.5	40.9	32.1	Left mPFC	8,9,10	Executive control of behavior [48]
F4	40.2	47.6	32.1	Right mPFC	8,9,10	Memory and decision making [49]
PZ	0.2	−62.1	64.5	PCC/Precuneus	7	Pain perception & goal processing [50]
P3	−39.5	−76.3	47.4	Left LPC	39,40	Theory of mind [51]
P4	38.8	−74.9	49.2	Right LPC	39,40	Recognition and working memory [52]
CP5	−62	−42	32	Left supramarginal cortex	40	Visuospatial processing [53]
CP6	66	−34	40	Right supramarginal cortex	40	Planning and motor imagery [54]

**Table 3:** ANOVA comparisons between partial directed coherence (PDC) values and social anxiety disorder (SAD) groups.

Band	Independent Variables	F	*p* Value	η2
Delta	SAD Groups	3.937	0.009	0.1
Theta	SAD Groups	2.389	0.069	0.05
Alpha	SAD Groups	3.766	0.001	0.1
Low beta	SAD Groups	0.410	0.196	0.04
High beta	SAD Groups	1.571	0.746	0.04

**Table 4:** A comparison between the current study and relevant studies in the literature.

Ref	Method	Network	No. Subjects	Main Findings
[20]	fMRI	DMN	84	Increased DMN activity in PCC and LPC.
[22]	fMRI	Salience network & DMN	12	DMN connectivity was not different between groups.
[75]	fMRI	DMN	8	SAD showed higher activation in the precuneus than HCs.
[76]	fMRI	DMN	40	The FC in the right precuneus had decreased in SAD patients as compared to HC.
[77]	EEG	DMN	47	SAD individuals showed a decrease of FC between mPFC and PCC.
This study	EEG	DMN	88	Enhanced EC between the DMN regions in SAD patients compared to HCs in resting-state.

## Data Availability

Data available on request due to privacy/ethical restrictions.

## Supplementary Materials

The following are available online at https://www.mdpi.com/article/10.3390/s21124098/s1, Figure S1: The distribution of the average absolute power for all SAD groups in different frequency bands, Figure S2: The distribution of the average absolute power for all SAD groups in different frequency bands ranging from 0 to 8, Figure S3: Relationship between the average PDC values and the regional DMN areas in different frequency bands, Figure S4: Correlation between DMN values and SIAS scores without dividing patients into different SAD conditions.

## Appendix A

Generally, a Multivariate autoregressive (MVAR) model with a number of cortical DMN regions (*m* regions) of EEG signals and order p is defined as follows:

(A1) $$X\left(t\right)=\overset{p}{\underset{r=1}{\sum }}A\;\left(r\right)\;X\;\left(t-r\right)+E\left(t\right),$$

where

(A2) $$\mathrm{Al}=\left[\begin{array}{ccc} a_{1}\left(l\right) & \cdots & a_{1n}\left(l\right)\\ \vdots & \cdots & \vdots \\ a_{n}\left(l\right) & \cdots & a_{nn}\left(l\right) \end{array}\right],$$

is the coefficient matrix at the time lag (*l*). X(*t*) represents the weight vector of *m* ROIs of EEG signals at time *t*, matrix A(*r*) indicates the *r*-th order AR parameters, and E(*t*) represents the measured error that is believed to be an independent Gaussian process with zero mean.

When the coefficients of the MVAR model are adequately calculated, A(*F*) is determined as follows:

(A3) $$A\left(F\right)=\overset{p}{\underset{r=1}{\sum }}A\left(r\right)e^{-i2\pi fr},$$

Therefore, the PDC value from channel j to channel i can be expressed as follows:

(A4) $${\mathrm{PDC}}_{\mathrm{ij}}\;\left(f\right)=\frac{A_{ij}\;{\left(f\right)}^{-}}{\sqrt{aj^{-H}\;\left(f\right){\mathrm{aj}}^{-}\left(f\right)}},$$

where, āi (*f*) (*i* = 1, 2, …*M*) represents the i-th column of the matrix Ā(*f*) and PDC_ij_ represents the directional influence and intensity of the information flow from channel j to channel i at a frequency of *f*. The PDC values were computed for each combination and used as an input feature for the classifiers. Features from these frequency bands were chosen to provide optimum accuracy with optimal order at *p* = 5–7 [80].
