# Supplementary material for: Analysis of Default Mode Network in Social Anxiety Disorder: EEG Resting-State Effective Connectivity Study
Source: Sensors (Basel). 2021 Jun 15;21(12):4098. doi: 10.3390/s21124098 (PMC8232236; doi:10.3390/s21124098)
Supplement: Supplementary file 1 [file sensors-21-04098-s001.zip › sensors-1141235-supplementary.pdf]

Supplementary Materiasl

Analysis of Default Mode Network in Social Anxiety Disorder: EEG Resting-State Effective Connectivity Study

Abdulhakim Al-Ezzi [1], Nidal Kamel [1,*], Ibrahima Faye [1] and Esther Gunaseli [2]

1 Department of Electrical and Electronic Engineering, Centre for Intelligent Signal and Imaging Research (CISIR), Universiti Teknologi PETRONAS, 32610 Seri Iskandar, Malaysia; abduhalezzy@yahoo.com (A.A.-E.); ibrahima_faye@utp.edu.my (I.F.)

2 Psychiatry Discipline Sub Unit, Universiti Kuala Lumpur, 31400 Perak, Malaysia; esther@unikl.edu.my

* Correspondence: nidalkamel2@hotmail.com

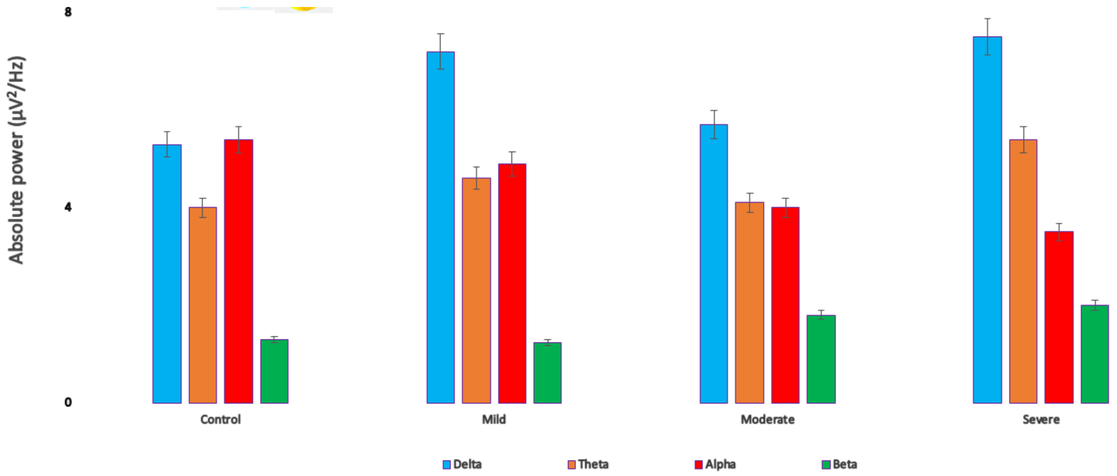

Figure S1. The distribution of the average absolute power for all SAD groups in different frequency bands.

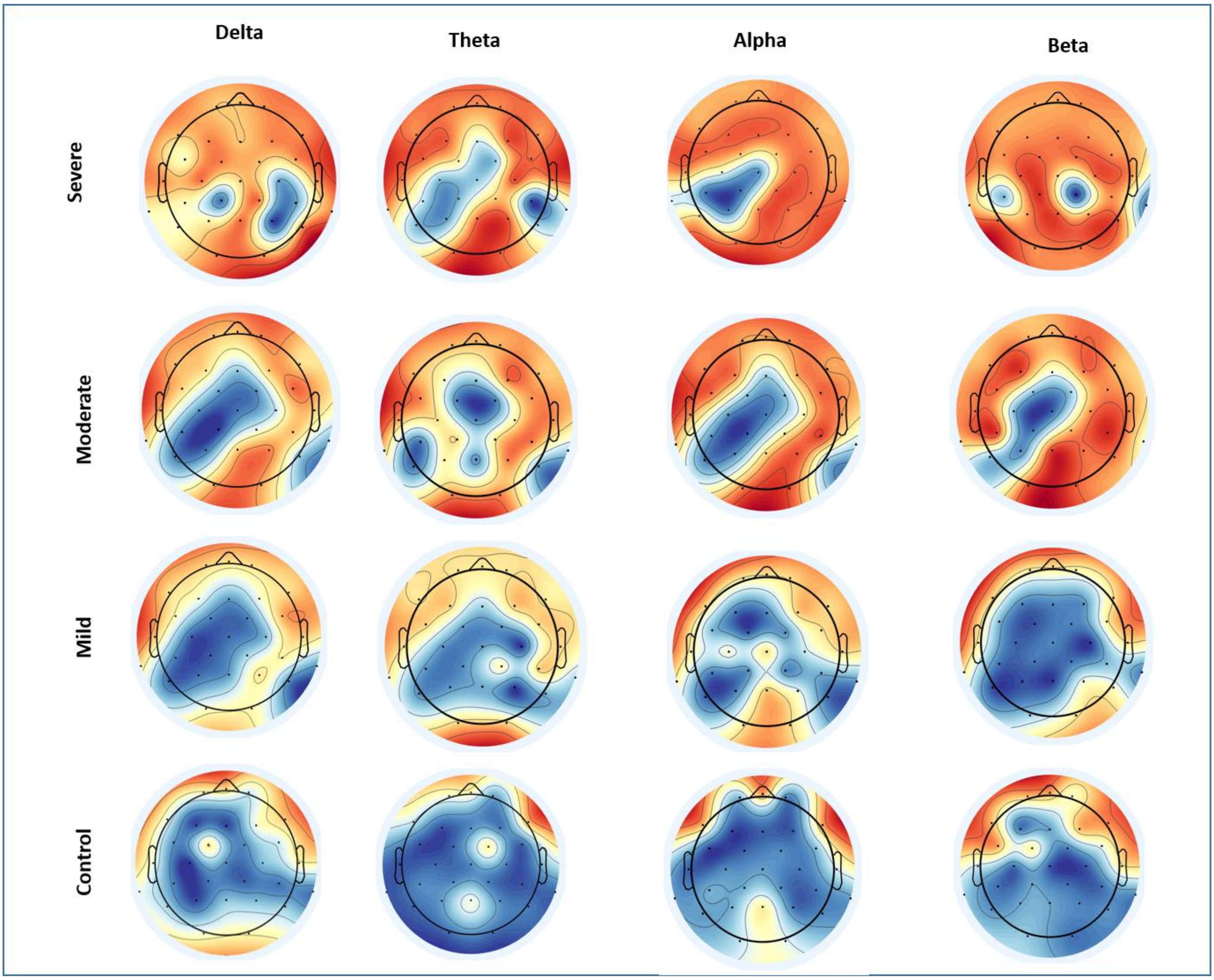

Figure S2. The distribution of the average absolute power for all SAD groups in different frequency bands ranging from 0 to 8.

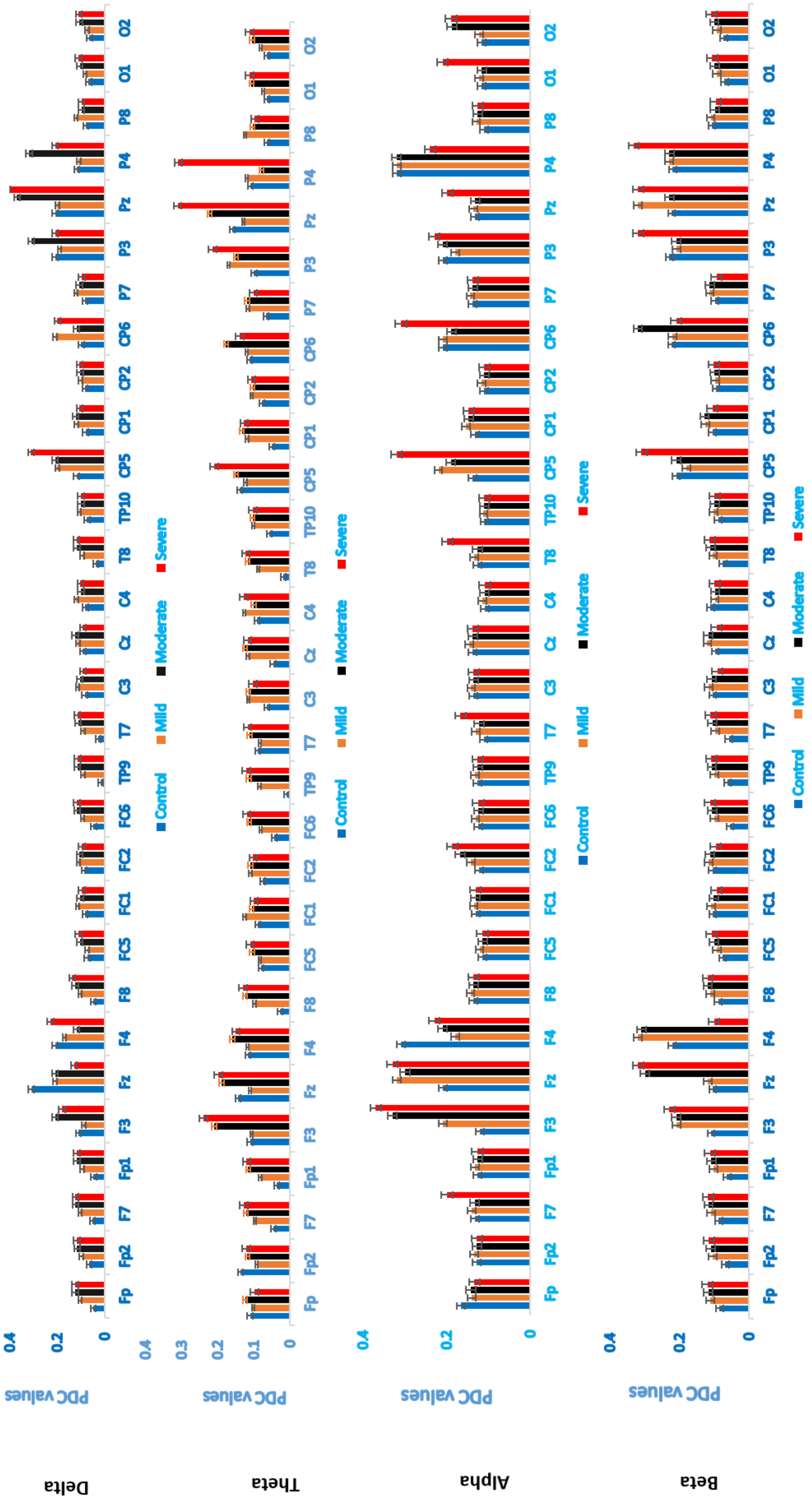

Figure S3. Relationship between the average PDC values and the regional DMN areas in different frequency bands.

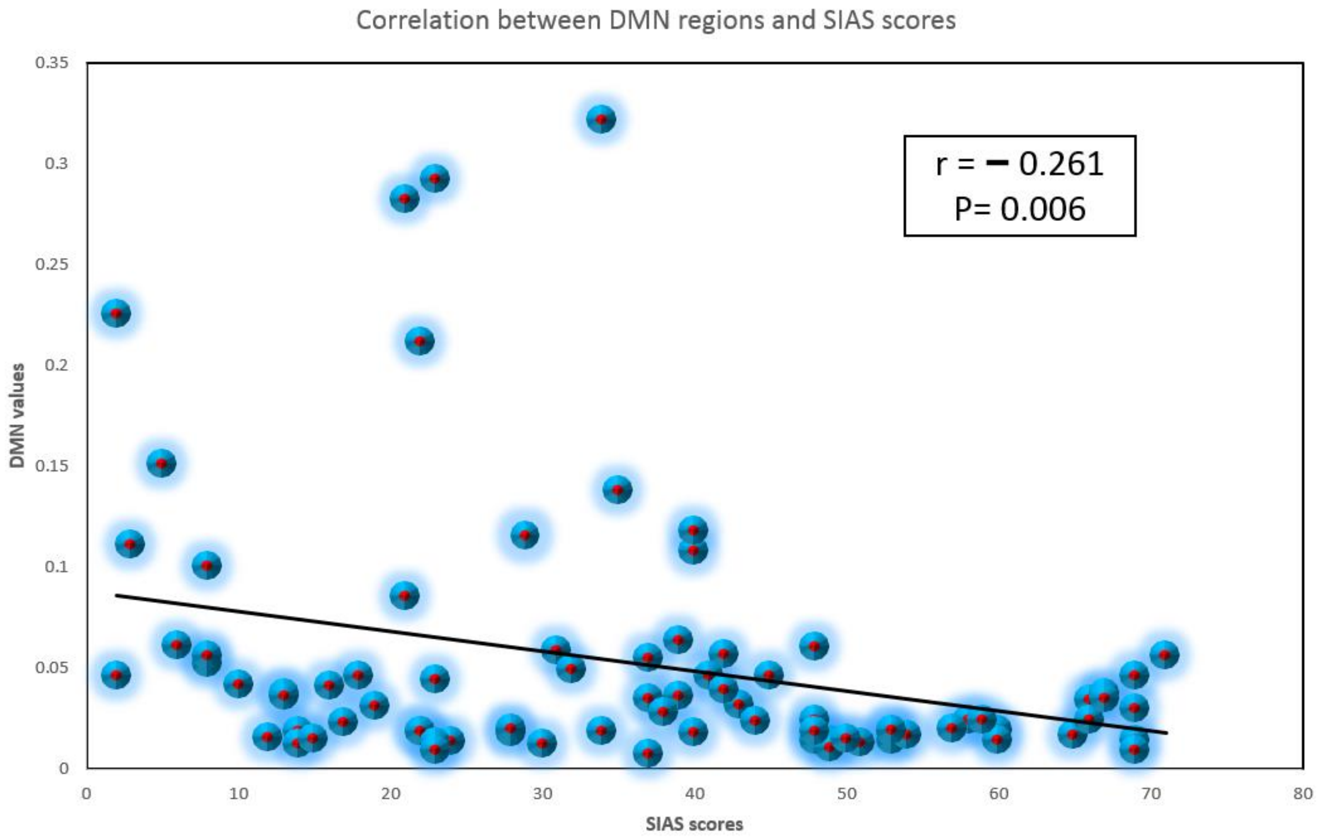

Figure S4. Correlation between DMN values and SIAS scores without dividing patients into different SAD conditions.
